# A rapid and sensitive method for the simultaneous analysis of aliphatic and polar molecules containing free carboxyl groups in plant extracts by LC-MS/MS

**DOI:** 10.1186/1746-4811-5-17

**Published:** 2009-11-25

**Authors:** Mario Kallenbach, Ian T Baldwin, Gustavo Bonaventure

**Affiliations:** 1 Max Planck Institute for Chemical Ecology, Department of Molecular Ecology, Hans Knöll Str. 8, 07745 Jena, Germany

## Abstract

**Background:**

Aliphatic molecules containing free carboxyl groups are important intermediates in many metabolic and signalling reactions, however, they accumulate to low levels in tissues and are not efficiently ionized by electrospray ionization (ESI) compared to more polar substances. Quantification of aliphatic molecules becomes therefore difficult when small amounts of tissue are available for analysis. Traditional methods for analysis of these molecules require purification or enrichment steps, which are onerous when multiple samples need to be analyzed. In contrast to aliphatic molecules, more polar substances containing free carboxyl groups such as some phytohormones are efficiently ionized by ESI and suitable for analysis by LC-MS/MS. Thus, the development of a method with which aliphatic and polar molecules -which their unmodified forms differ dramatically in their efficiencies of ionization by ESI- can be simultaneously detected with similar sensitivities would substantially simplify the analysis of complex biological matrices.

**Results:**

A simple, rapid, specific and sensitive method for the simultaneous detection and quantification of free aliphatic molecules (e.g., free fatty acids (FFA)) and small polar molecules (e.g., jasmonic acid (JA), salicylic acid (SA)) containing free carboxyl groups by direct derivatization of leaf extracts with Picolinyl reagent followed by LC-MS/MS analysis is presented. The presence of the N atom in the esterified pyridine moiety allowed the efficient ionization of 25 compounds tested irrespective of their chemical structure. The method was validated by comparing the results obtained after analysis of *Nicotiana attenuata *leaf material with previously described analytical methods.

**Conclusion:**

The method presented was used to detect 16 compounds in leaf extracts of *N. attenuata *plants. Importantly, the method can be adapted based on the specific analytes of interest with the only consideration that the molecules must contain at least one free carboxyl group.

## Background

The analysis of low abundant signalling molecules such as phytohormones (e.g., jasmonic acid (JA), salicylic acid (SA) and abscisic acid (ABA)) and intermediates of metabolic pathways (e.g., free fatty acids (FFA), oxygenated forms of fatty acids) in plants is an important tool to understand how plants grow, develop and respond to stress conditions. For high-throughput biochemical phenotyping of, for example, wild-type or genetically modified plants grown under diverse conditions, it is essential to develop methods for the rapid, simultaneous and reliable quantitative analysis of a broad range of molecules.

The use of tandem mass spectrometry (MS/MS) coupled to liquid chromatography (LC) is ideal for the analysis of complex mixtures of compounds which are commonly found in biological matrices such as plant tissues. One of the advantages of LC-MS/MS is that separation and structural elucidation of compounds can be achieved in a continuous manner without the need for purification or derivatization steps. Another advantage of LC-MS/MS is the use of tandem MS, in which a precursor ion is mass-selected by mass analyzer 1, focused into a collision region preceding a second mass analyzer (collision chamber), and their mass fragments analyzed in a third mass analyzer [1]. The capacity to perform multi reaction monitoring (MRM) has the advantage of rapid and sensitive detection of several compounds even if they show similar retention times during LC.

Ionization by electrospray (ESI) is one of the most widely used tools for LC-MS/MS analysis, and the ions can be selectively monitored in negative and positive mode [2]. In the ESI negative mode, analysis of small molecules containing free carboxyl groups, yields mainly the ion [M-H]^-^, corresponding to their carboxylate anion. However, the efficiency of formation of carboxylate anions differs widely among compounds depending on their chemical structure [3]. For example, while small molecules containing free carboxyl groups and high numbers of heteroatoms such as the phytohormones JA, SA and ABA or aromatic substances are ionized efficiently by ESI [4-6], aliphatic molecules such as FFAs are relatively poorly ionized by this technique, in particular, as the aliphatic chain becomes longer and the degree of saturation higher [7]. This lower efficiency of carboxylate anion formation restricts the analysis of aliphatic molecules, specially, when small amounts of tissue are available for analysis (e.g., embryos, pollen, tissue sections obtained by laser micro-dissection).

Analytical methods to quantify aliphatic molecules are laborious, involving enrichment steps using chromatographic techniques such as thin layer chromatography (TLC), solid phase extraction (SPE) or LC previous to derivatization and gas chromatography (GC) for separation and analysis [8]. One strategy to improve the sensitivity of analysis of aliphatic molecules containing carboxyl groups by LC-MS/MS is the use of chemical derivatization that generates a strong ion in the ESI source [8]. Among these, the generation of pyridinium compounds has many advantages [9]. The presence of the N atom in the pyridine moiety allows for the efficient ionization of the compounds [9] and due to the mild conditions used to generate the Picolinyl ester intermediates, analysis of sensitive molecules containing conjugated double bonds is possible [10]. Moreover, since ester bonds are resistant to the conditions used to generate Picolinyl ester derivatives, esterified fatty acids do not interfere with the analysis [10].

In this study, a method for the simultaneous determination of aliphatic molecules (e.g., FFAs and their oxygenated derivatives) and more polar (phytohormones, phenolics) compounds by using Picolinyl ester derivatives of leaf extracts coupled with LC-MS/MS is presented. The method was applied for the analysis of leaves of *N. attenuata *plants before and after wounding and elicitation by the insect elicitors fatty acid-amino acid conjugates (FACs)[11]; which are treatments known to stimulate the production of a large number of phytohormones and secondary metabolites [12].

## Results and Discussion

### Method development

**Figure 1:**
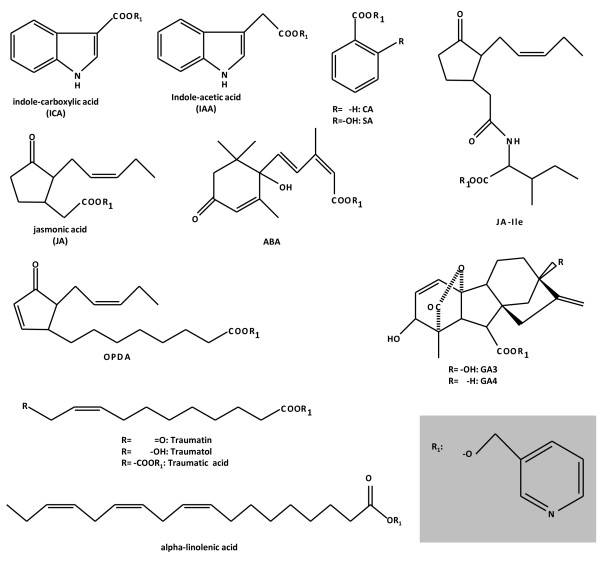
**Examples of compounds analyzed as their Picolinyl ester derivatives by LC-MS/MS**.

**Figure 2:**
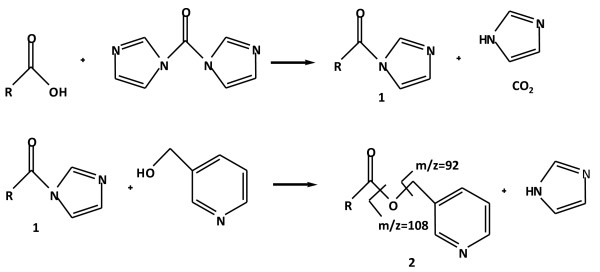
**Reaction mechanisms for the formation of Picolinyl ester derivatives of carboxylic acids**. The carboxyl group is first activated by reaction with 1,1'-carbidiimidazole to form the active amid 1. 1 is then transesterified with 3-(hydroxymethyl)-pyridine to form the Picolinyl ester derivative 2. After collision induced dissociation (CID), the major ion fragments obtained are *m/z *= 92 and *m/z *= 108.

Commercial chemical standards (Table 1) were first derivatized to their respective Picolinyl esters using the mild method proposed in [10]. Some examples of the molecules used are shown in Figure 1. This method allows the quantitative derivatization of free carboxyl group-containing molecules within 10 min using conditions that preserve sensitive molecules containing conjugated double bonds. The first step in the reaction involves the activation of the carboxyl group with 1,1'-carbonyldiimidazole to form an active carbimidazol amid (**1**, Figure 2). The second step involves the reaction of **1 **with 3-(hydroxymethyl)-pyridine to form the corresponding β-Picolinyl ester (**2**, Figure 2).

**Table 1:** List of standards used for matrix free and matrix adapted calibration.

				Calibration^2^		Calibration^3^	
				
Number	Substance	[M+H]^+^	Retention time [min]	Linearity (r)^§^	LOD [pg/μL]	Linearity (r)^§^	LOD [pg/μL]
1	^13^C_6_-jasmonic acid-isoleucine (^13^C_6_-JA-Ile)	421	6.86	1.00	20.60	0.99	116
2	Jasmonic acid-isoleucine (JA-Ile)	415	6.86	1.00	20.80	0.99	117
3	^2^H_4_-salicylic acid (^2^H_4_-SA)	234	8.77	0.98	24.40	0.98	167
4	Salicylic acid (SA)	230	8.77	0.97	24.80	0.98	165
5	^2^H_6_-abscisic acid (^2^H_6_-ABA)	362	9.01	0.98	19.40	0.98	163
6	Abscisic acid (ABA)	356	9.02	0.99	18.70	0.98	165
7	Indole-3-carboxylic acid (ICA)	253	9.08	0.93	41.30	0.91	355
8	Royal jelly acid (Tr IS)	278	9.26	0.99	12.00	0.98	181
9	Cinnamic acid (CA)	240	9.34	0.99	23.90	0.97	203
10	Jasmonic acid (JA)	302	9.43	0.98	19.60	0.98	155
11	Traumatol	306	9.85	0.99	12.90	0.98	174
12	Indole-3-acetic acid (IAA)	267	9.94	0.98	19.30	0.99	297
13	Traumatin	304	9.96	1.00	5.30	0.98	168
14	9,10-^2^H_2_-dihydro-jasmonic acid (D_2_-JA)	306	10.13	1.00	11.30	0.98	180
15	Traumatic acid	320	10.15	0.99	11.20	0.96	250
16	Hexadecatrieonic acid (16:3)	342	10.29	0.99	17.00	0.99	132
17	(9 S, 13 S)-12-oxo-phytodienoic acid (OPDA)	384	10.89	1.00	10.30	0.98	147
18	Hexadecadienoic acid (16:2)	344	10.93	0.99	14.90	0.98	147
19	Gibberellin A_3 _(GA_3_)	438	11.27	0.93	42.30	0.96	246
20	^2^H_2_-OPC 8:0	388	11.59	0.94	*^1^	0.96	*^1^
21	Linolenic acid (18:3)	370	12.83	1.00	7.80	1.00	70
22	Hexadecenoic acid (16:1)	346	13.20	0.99	19.00	0.99	95
23	Heptadecenoic acid (17:1)	360	13.78	1.00	4.10	0.97	195
24	Linoleic acid (18:2)	372	14.22	0.97	8.60	0.96	123
25	Hexadecanoic acid (16:0)	348	14.53	1.00	5.20	0.99	114
26	Octadecenoic acid (18:1)	374	14.86	0.99	14.70	0.99	94
27	Heptadecanoic acid (17:0)	362	16.09	1.00	7.50	0.99	119
28	Stearic acid (18:0)	376	17.59	1.00	7.90	0.99	117

*^1 ^Amount of standard unknown.

^2 ^Matrix free calibration

^3 ^Matrix adapted calibration

^§^Standard calibration curves were generated by injecting 10 μL of 100, 250, 375, 500, 750, 1000 pg/μL of the different standards (*n *= 3).

As mentioned in the background section, the formation of Picolinyl esters by this method is restricted to free carboxyl groups [10]. To confirm the absence of hydrolysis of ester bonds from esterified fatty acids, 10 μg of commercial glycerolipids (monogalactosyldiglycerol: MGDG, phosphatidylcholine: PC and phosphatidylglycerol: PG) were first purified by TLC and then subjected to the reaction. No free fatty acids could be detected (data not shown and see below for the methodology used for detection), confirming that the reaction does not induce the hydrolysis of esterified fatty acids.

Analysis of the Picolinyl ester derivatives of the commercial standards was first accomplished by their direct injection into the MS-interface to determine their [M+H]^+ ^parent ion and their MS/MS fragmentation pattern by collision induced dissociation (CID) using increasing voltage energies. The third mass analyzer was set in the scan mode for ions with *m/z *between 50 and 500. Two major fragments were generated, *m/z *= 92 and *m/z *= 108, resulting from the loss of the methyl-pyridine fragment and the hydroxymethyl-pyridine fragment, respectively (Figure 2). The ion *m/z *= 92 gave the strongest intensity at a collision energy of -25.5 V and therefore the [M+H]^+ ^> 92 *m/z *transition was used for specific detection of Picolinyl ester derivatives.

**Figure 3:**
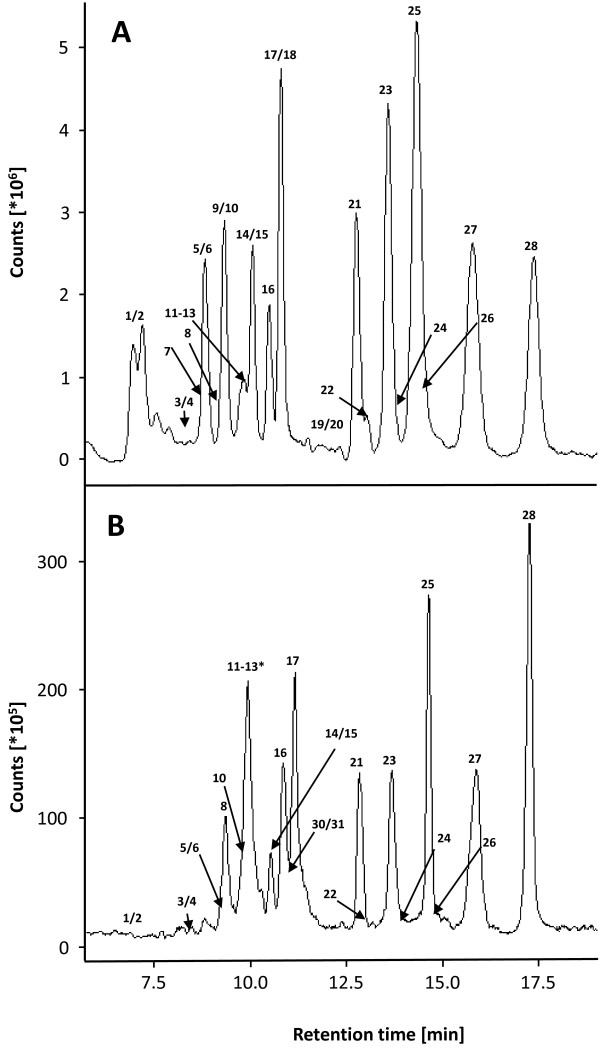
**Example of chromatograms of Picolinyl ester derivatives from a standard mixture and a *N. attenuata *leaf extract**. A, Chromatogram (TIC) of a mix of derivatized commercial standards. B, Chromatogram (TIC) of a derivatized *N. attenuata *leaf extract after 60 min of FAC elicitation. *Analytes 11 to 13 are overlaid by a peak corresponding to an unknown compound in the leaf extract. Peaks are numbered according to Table 1.

The chromatographic separation of Picolinyl ester derivatives was performed on a reverse-phase column using acidic water and methanol as solvents in a gradient mode. In this case, the LC method was optimized for the analysis in plant extracts of small polar molecules and aliphatic molecules containing no more than 18 carbons. After a pre-run of 1.5 min, all substances of interest eluted from the column in 18.5 min. An additional post run of 6.5 min was added for column conditioning for a final run time of 25 min. An example of chromatograms (total ion current, TIC) for derivatized commercial standards and a derivatized leaf extract from *N. attenuata *is shown in Figures 3A and 3B, respectively.

Mixtures of derivatized commercial standards ranging from 100 to 1000 pg/μL were first used to determine their linear range of detection and their limit of detection (LOD). These concentrations were in the same range as the endogenous compounds quantified in derivatized leaf extracts (see below). Within this range, most analytes presented a linear response (concentration vs. area) with r values higher than 0.97 with the exception of ICA, GA_3 _and ^2^H_2_-OPC 8:0 which presented r values between 0.93 and 0.94 (Table 1). The LODs, calculated based on the calibration plot method, were between 5 and 42 pg/μL (Table 1). To determine the injection precision, each derivatized commercial standard was injected 10 times at different concentrations and the coefficient of variation (CV) was calculated. For all compounds, the CV values were less than 0.1 for all concentrations tested (Additional file 1, Table S1).

To determine matrix suppression effects in a leaf extract, mixtures of derivatized standards were spiked at different concentrations in underivatized leaf extracts of *N. attenuata *plants and the linear range of detection and LOD were calculated (Table 1). In this case, the linearity of the response (r value, concentration vs. area) was similar to that presented by the derivatized standards in pure solvent, however, the LOD values were increased for all standards to amounts between 70 and 350 pg/μL.

### Extraction and analysis of *N. attenuata *leaves

The extraction of polar and lipophilic compounds from *N. attenuata *leaves was performed as indicated by [13] with the modifications adopted by [14]. Additional modifications were included to increase the number of samples that can be processed simultaneously and to reduce the amount of sample material necessary for analysis (see Methods section).

The extraction method was first validated by performing 10 biological replicates of *N. attenuata *non-elicited and FAC-elicited leaves after 60 min of the treatment. For each replicate, 300 mg of leaf tissue were extracted, derivatized and analyzed by LC-MS/MS (Additional file 1, Table S2). The standard deviations were below 10% of the average values for all detectable compounds. To determine the extraction recovery rate, the residual leaf material obtained after the first extraction was re-extracted, derivatized and analyzed. The recovery rates were higher than 98% for all molecules tested (Additional file 1, Table S2). Sample stability was tested by re-analyzing the derivatized leaf extracts after two days of the first analysis (samples were kept at 10°C). The amounts of all compounds did not differ significantly between the first and second analyses (paired *t*-test, data not shown). The CV was analyzed for the detectable compounds in derivatized leaf extracts (analyzed the same day of extraction) from FAC-elicited leaves (60 min after the treatment). The CV values of two-day old extracts (kept at 10°C) were also analyzed. The CV values were below 0.1 for all detectable compounds (Additional file 1, Table S1). Finally, the efficiency of derivatization was evaluated by the analysis of the respective free compounds in derivatized leaf extracts (60 min after FAC elicitation). No free molecules were detected, corroborating that the derivatization is quantitative [10].

To validate the results obtained with the method presented in this study, 300 mg of non-elicited leaves of *N. attenuata *plants and leaves wounded and elicited with FAC (30 and 60 min) were analyzed with the present method and additionally with well established analytical methods for aliphatic compounds and phytohormes. Aliphatic compounds (free fatty acids in this case) were analyzed by performing separation by TLC and GC-MS analysis of their methyl-ester derivatives (see Methods section) and phytohormones were analyzed by LC-MS/MS using underivatized leaf extracts [6].

A total of 25 compounds were analyzed, including FFAs, derivatives and intermediates of the lipoxygenase pathway (e.g., OPDA, dnOPDA, OPCs) and some phytohormones related to stress responses or growth (e.g., JA, SA, ABA, JA-Ile, GAs, IAA). The analysis showed that 16 compounds could be detected in derivatized *N. attenuata *leaf extracts after elicitation. ICA, CA, traumatol, dnOPDA, IAA, 16:2, GA_3_, OPC-8:0 and GA_4 _could not be detected by any of the methods used (Table 2). The phytohormones IAA, GA_3_, and GA_4 _accumulate to low levels in leaf tissue and their detection usually requires purification steps. Thus, these molecules were most likely below their LOD (Table 1). Likewise, levels of free 16:2, CA, OPC-8:0, dnOPDA and traumatol could be either below their LOD or in some cases (CA and dnOPDA) absent in *N. attenuata *leaves. JA-Ile and traumatic acid could not be detected by the present method in non-elicited tissue however they were detected in low amounts by analysis of underivatized extracts (Table 2). Nevertheless, as the amount of these molecules increased after FAC elicitation, they became detectable in their Picolinyl ester forms (Table 2). In contrast, OPC-4:0 and OPC-6:0 could be detected only by the present method after FAC elicitation but not by the analysis of underivatized extracts. For the remaining compounds, a good correlation was observed between the methods used (Table 2).

**Table 2:** Quantification of Picolinyl ester derivatives of *N. attenuata *leaf extracts and comparison with two additional analytical methods.

			Treatments									
			
			0 min		30 min				60 min			
			
					wound		FAC		wound		FAC	
	
Substance	RT	[M+H]^+^	Picol. esters	Method 2 or 3*	Picol. esters	Method 2 or 3*	Picol. esters	Method 2 or 3*	Picol. esters	Method 2 or 3*	Picol. esters	Method 2 or 3*
			ng gFW^-1 ^(± SE)	ng gFW^-1 ^(± SE)	ng gFW^-1 ^(± SE)	ng gFW^-1 ^(± SE)	ng gFW^-1 ^(± SE)	ng gFW^-1 ^(± SE)	ng gFW^-1 ^(± SE)	ng gFW^-1 ^(± SE)	ng gFW^-1 ^(± SE)	ng gFW^-1 ^(± SE)
JA-Ile	6.86	415	**-**	**1.1** (± 0.14)	**39** (± 4.0)	**46** (± 4.8)	**92** (± 6.0)	**81** (± 2.0)	**83** (± 9.0)	**97** (± 4.0)	**158** (± 16)	**163** (± 19)
SA	8.77	230	**193** (± 31)	**173** (± 23)	**203** (± 24)	**212** (± 19)	**182** (± 15)	**196** (± 11)	**216** (± 22)	**189** (± 17)	**116** (± 18)	**129** (± 22)
ABA	9.01	356	**257** (± 7)	**225** (± 8.0)	**192** (± 35)	**246** (± 9.0)	**274** (± 16)	**232** (± 14)	**264** (± 6.0)	**242** (± 10)	**277** (± 12)	**270** (± 14)
ICA	9.08	253	**-**	**-**	**-**	**-**	**-**	**-**	**-**	**-**	**-**	**-**
CA	9.34	240	**-**	**-**	**-**	**-**	**-**	**-**	**-**	**-**	**-**	**-**
JA	9.43	302	**137** (± 20)	**152** (± 23)	**937** (± 150)	**845** (± 95)	**1845** (± 125)	**1586** (± 110)	**1564** (± 180)	**1326** (± 42)	**3820** (± 350)	**3149** (± 223)
Traumatol	9.85	306	**-**	**-**	**-**	**-**	**-**	**-**	**-**	**-**	**-**	**-**
dnOPDA	9.85	356	**-**	**-**	**-**	**-**	**-**	**-**	**-**	**-**	**-**	**-**
IAA	9.94	267	**-**	**-**	**-**	**-**	**-**	**-**	**-**	**-**	**-**	**-**
Traumatin	9.96	304	**11.3** (± 5.6)	**14.4** (± 7.8)	**17.1** (± 4.8)	**15.2** (± 2.3)	**14.6** (± 3.4)	**12.4** (± 2.7)	**13.8** (± 1.9)	**15** (± 5.2)	**10.7** (± 3.6)	**12.2** (± 1.6)
Tr. acid**	10.15	320	**-**	**5** (± 0.96)	**19.6** (± 3.2)	**24.3** (± 6.7)	**23.8** (± 6.1)	**20.4** (± 4.8)	**17.1** (± 2.5)	**19.6** (± 2.3)	**20.9** (± 1.9)	**21.4** (± 4.2)
OPC4:0	10.46	330	**-**	**-**	**-**	**-**	**12.6** (± 8.1)	**-**	**10.3** (± 4.9)	**-**	**18.5** (± 4.7)	**-**
OPC6:0	10.67	358	**-**	**-**	**-**	**-**	**5.3** (± 2.9)	**-**	**8.6** (± 3.5)	**-**	**11.2** (± 5.6)	**-**
OPDA	10.89	384	**107** (± 60)	**91** (± 53)	**323** (± 47)	**386** (± 45)	**291** (± 11)	**322** (± 15)	**180** (± 45)	**184** (± 15)	**301** (± 31)	**313** (± 3.0)
GA_3_	11.27	438	**-**	**-**	**-**	**-**	**-**	**-**	**-**	**-**	**-**	**-**
OPC8:0	11.41	386	**-**	**-**	**-**	**-**	**-**	**-**	**-**	**-**	**-**	**-**
GA_4_	11.99	424	**-**	**-**	**-**	**-**	**-**	**-**	**-**	**-**	**-**	**-**

18:3	12.83	370	**446** (± 38)	**398** (± 24)	**480** (± 53)	**497** (± 37)	**523** (± 30)	**497** (± 42)	**605** (± 20)	**596** (± 31)	**476** (± 33)	**424** (± 31)
16:1	13.2	346	**52** (± 9)	**26** (± 23)	**41** (± 3.0)	**22** (± 20)	**48** (± 12)	**51** (± 32)	**91** (± 26)	**147** (± 96)	**103** (± 13)	**121** (± 83)
16:2	10.93	344	**-**	**-**	**-**	**-**	**-**	**-**	**-**	**-**	**-**	**-**
16:3	10.29	342	**45.6** (± 12)	**-**	**41.1** (± 2.0)	**28.2** (± 23.0)	**64.1** (± 19)	**40.5** (± 21)	**130.2** (± 10)	**80.9** (± 39)	**37.2** (± 3.0)	**-**
18:2	14.22	372	**245** (± 16)	**190** (± 17)	**251** (± 8.0)	**189** (± 13)	**316** (± 45)	**398** (± 23)	**377** (± 43)	**415** (± 26)	**333** (± 209)	**367** (± 25)
16:0	14.53	348	**21100** (± 3000)	**22300** (± 4150)	**16600** (± 930)	**14100** (± 2300)	**18600** (± 2800)	**15400** (± 4700)	**23200** (± 3200)	**21600** (± 3000)	**14600** (± 900)	**15600** (± 3300)
18:1	14.86	374	**625** (± 47)	**502** (± 66)	**603** (± 22)	**572** (± 72)	**805** (± 59)	**716** (± 28)	**791** (± 84)	**778** (± 47)	**894** (± 36)	**813** (± 51)
18:0	17.59	376	**46800** (± 12900)	**44600** (± 7300)	**47900** (± 1200)	**33400** (± 9300)	**49000** (± 5100)	**36700** (± 4600)	**45700** (± 6200)	**41300** (± 3300)	**39400** (± 5300)	**33000** (± 2700)

Quantification of compounds was performed in triplicate (*n *= 3)

-: below limit of detection under the conditions tested

* Method 2: Analysis of underivatized molecules by LC-MS/MS, this method was used to quantify compounds in the upper list section

* Method 3: Analysis of methyl esters of FFA (FAMES) by GC-MS

** Tr. acid: Traumatic acid

**Figure 4:**
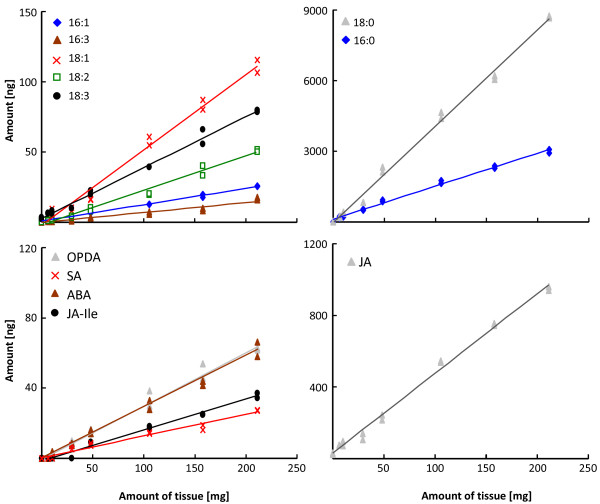
**Example of the linear correlation between the amounts of leaf tissue extracted from FAC elicited *N. attenuata *leaves and the amounts of compounds detected**. Different amounts of leaf tissue (5 to 250 mg; fresh weight) from *N. attenuata *plants were collected after 60 min of FAC elicitation. Leaf material was extracted, derivatized and analyzed by LC-MS/MS. Two biological replicates were performed for each amount of tissue.

Finally, increasing amounts of leaf material (5 to 200 mg; fresh weight) were also extracted from *N. attenuata *plants after 60 min of FAC elicitation to determine the range of tissue amounts in which a linear correlation with the amounts of detectable compounds was observed. The results showed a linear correlation between the amount of 11 compounds and the amount of leaf material extracted (Figure 4), indicating that as little as 5 mg of leaf tissue was sufficient for the reliable quantification of these molecules. In the case of JA-Ile, at least 50 mg of leaf tissue were required for detection while for OPC-6:0, OPC-4:0, traumatin and traumatic acid more than 100 mg of tissue were required (Additional file 1, Table S3).

## Conclusion

A method was developed that enables the rapid, specific and simultaneous analysis of aliphatic compounds such as free fatty acids and small polar compounds such as phytohormones in *N. attenuata *leaves. Although in this study only a selected number of molecules were tested, the method can be adapted to the needs of the investigator. This method may prove useful in cases in which tissue amounts are limiting (e.g., embryos, ovules, pollen or specific tissue sections obtained by laser micro-dissection) and multiple analytical techniques cannot be used. Additionally, the rapid extraction procedure without additional purification steps may also facilitate the analysis of multiple samples quickly, making it compatible with high-throughput biochemical screenings.

## Methods

### Plant material and treatments

Seeds of the 30^th ^generation of an inbred line of *Nicotiana attenuata *plants were used as the wild-type genotype (WT) in all experiments. Plants were grown as described in [15]. For all experiments, leaves at nodes +1 of rosette stage (40-day old) plants were used. Wounding was performed by rolling three times a fabric pattern wheel on each side of the midvein. For FAC treatment, the wounds were immediately supplied with 20 μL of synthetic *N*-linolenoyl-glutamic acid (18:3-Glu; 0.03 nmol/μL in 0.02% (v/v) Tween-20/water). Leaf tissue was collected at 30 and 60 min after the treatments and frozen immediately in liquid nitrogen for subsequent analysis. Non-elicited leaf tissue was collected without any previous treatment.

### Chemicals

Chloroform, dichloromethane, methanol and hexane were from VWR (International GmbH, Darmstadt, Germany). JA, SA, ABA, IAA, ICA, traumatic acid, 1, 1'-carbonyldiimidazole, 3-(hydroxymethyl)-pyridine and fatty acids were from Sigma (Taufkirchen, Germany). OPDA was from Cayman Chemicals (Ann Arbor, MI). Cinnamic acid was from Merck KGaA (Darmstadt, Germany). Gibberellins was from Carl Roth GmbH (Karlsruhe, Germany). Traumatin was from Larodan Chemicals (Malmö, Sweden). Traumatol was synthesized by the reduction of traumatin with NaBH_4 _using standard conditions and ^2^H_2_-OPC 8:0 was synthesized by deuteration of OPDA using standard conditions.

### Leaf extraction

Extractions were performed according to [13] with the modifications adopted by [14]. Depending on the experiment, different amounts of frozen leaf material were homogenized in 2 mL microcentrifuge tubes (Eppendorf, Hamburg, Germany) containing 2 steel beads (ASK, Korntal-Muenchingen, Germany) by grinding in a Genogrinder (SPEX Certi Prep, Metuchen, NJ) for 30 sec at 1300 strokes min^-1^. After homogenization, samples were extracted with 1 ml of 10/10/1/1 (v/v/v/v) chloroform/methanol/acetic acid/water spiked with internal standards (0.5 μg heptadecanoic acid, 0.4 μg [^2^H_2_]-JA, [^2^H_4_]-SA, [^13^C_6_]-Ja-Ile, [^2^H_6_]-ABA, royal jelly acid). After vortexing for 10 min, the phases were separated by centrifugation at 4°C for 10 min. The organic phase was transferred into a fresh tube and the leaf material re-extracted with 1 ml 5/5/1 (v/v/v) chloroform/methanol/water. After centrifugation, the organic phases were combined, evaporated to dryness under a gentle stream of nitrogen and reconstituted in 0.2 mL of dry dichloromethane for subsequent derivatization. Quantification was made based on the internal standards added and calibration curves.

### Picolinyl ester derivatization

Formation of Picolinyl ester derivatives of free carboxylic acids was performed as described in [10]. Briefly, 0.2 mL of leaf extract in dichloromethane were mixed with 0.1 mL of freshly prepared 1% (w/v) 1, 1'-carbonyldiimidazole/dichloromethane. After 1 min, 0.2 mL of freshly prepared 0.1/1/1 (v/v/v) 3-(hydroxymethyl)-pyridine/dichloromethane/triethylamine containing catalytic amounts of 4-(1-pyrrolidinyl)-pyridine were added and the mixture heated for 10 min at 37°C. The reaction was stopped by the addition of 25 μL of acetic acid. Samples were dried under a stream of nitrogen and 0.5 mL of water were added. Picolinyl ester derivatives were extracted twice with 0.5 mL hexane, the solvent evaporated under a stream of nitrogen and the samples reconstituted in 70/30 (v/v) methanol/water for LC-MS/MS analysis.

### LC-MS/MS analysis

Picolinyl ester derivatives were analyzed in an LC-MS/MS system (Varian 1200 Triple-Quadrupole-LC-MS system; Varian, Palo Alto, CA, USA http://www.varianinc.com). Ten μL of the sample were injected onto a ProntoSIL^® ^column (C18; 5 μm, 50 × 2 mm, Bischoff, Germany; http://www.bischoff-chrom.de) attached to a precolumn (C18, 4 × 2 mm, Phenomenex, USA). As mobile phases 0.05%/1% (v/v/v) formic acid/methanol/water (solvent A) and methanol (solvent B) were used in a gradient mode with the following conditions: time/concentration (min/%) for B: 0.0/15; 1.5/15; 4.5/98; 19.5/98; 20.5/15; 25.0/15; time/flow (min/mL): 0.0/0.4; 1.0/0.4; 1.5/0.2; 18.5/0.2; 19.5/0.4; 25/0.4. To minimize contaminations the solvent eluting from the column was injected into the mass spectrometer only between 1.5 and 18.5 min. Between 0 and 1.5 min and 18.5 and 25 min a mixture of 1/1 (v/v) methanol/water was injected to flush the MS/MS system. The MS was used in ion positive mode and ions detected using multiple reaction monitoring (MRM) and their respective *m/z *transitions [M+H]^+ ^> 92 (Table 1) after collision induced fragmentation with argon gas under -25.5 V collision energy. For ionization, the needle was set at 5000 V and the drying gas (nitrogen) at 300°C and 20 psi (housing 50°C). The detector was set at 1800 V. Samples were kept at room temperature (RT) and separation was also performed at RT. Analysis of underivatized phytohormones from crude leaf extracts was performed as previously described [6].

### Analysis of FFA by GC-MS

FFA extraction was performed according to [14]. Heptadecanoic acid (0.5 μg per sample) was added as internal standard for quantification. After extraction, samples were reconstituted in 0.2 mL of chloroform and lipids were separated on Partisil^® ^K6 silica plates (Whatman, Dassel, Germany) which were developed with 70/30/1 (v/v/v) hexane/diethyl ether/acetic acid. Commercial FFAs were used as standards and plates were stained with 2% (v/v) 2'-7'-dichlorofluorescin/methanol and lipids were visualized under UV light. FFA were eluted from the silica with 3 mL of 2/1 (v/v) chloroform/methanol and methylated in 1% (v/v) H_2_SO_4_/methanol for 1 h at 75°C. Fatty acid methyl esters were extracted with hexane and analyzed in a Varian CP-3800 GC coupled with a Varian Saturn 3800 ion trap MS in electron ionisation (EI; 70 eV) mode (Varian, Palo Alto, CA). One μl of the sample was injected in splitless mode on a DB-WAX column (30 m × 0.25 mm I.D., 0.25 μm film thickness, Agilent, Boeblingen, Germany) with helium at a constant flow of 1 mL min^-1 ^as the carrier gas. The injector was at 230°C. The oven temperature program was: 130°C for 5 min, 220°C at 3.0°C/min, 5°C/min ramp to 240°C and hold for 1 min. EI spectra were recorded on Scan mode from 40 to 400 amu. Quantification was performed in the linear range of detection and based on calibration curves generated with increasing concentrations of commercial FAMES mixes (Matreya, Pleasant Gap, PA) and the IS (17:0).

## List of abbreviations

GC-MS: gas chromatography-mass spectrometry; ESI: electrospray ionization; MRM: multi reaction monitoring; FFA: free fatty acids; IAA: indole-3-acetic acid; IBA: indole-3-butyric acid; ICA: indole-3-carboxylic acid; ABA: abscisic acid; JA: jasmonic acid; JA-Ile: jasmonyl-isoleucine; OPDA: 12-oxo-phytodienoic acid; dnOPDA: dinor-OPDA; SA: salicylic acid; Traumatic acid: 2E-dodecendioic acid; Traumatin: 12-oxo-10*E*-dodecenoic acid; Traumatol: 12-hydroxy-9Z-dodecenoic acid; Royal jelly acid: 10-hydroxy-*trans*-2-decenoic acid; GA: gibberellin.

## Competing interests

The authors declare that they have no competing interests.

## Authors' contributions

MK carried out the experiments and drafted the manuscript. ITB participated in the design and coordination of the study and helped to draft the manuscript. GB conceived of the study, participated in its design and coordination and helped to draft the manuscript. All authors read and approved the final manuscript.

## Supplementary Material

Additional file 1 **Table S1**: Coefficients of variation (CV) of replicate measurements for standard mixtures at different concentrations and leaf sample. **Table S2**. Analysis of derivatized *N.attenuata *leaf extracts after multiple extractions and calculation of recovery rates. **Table S3**: Analysis of different amounts of *N. attenuata *leaf material. Click here for file
