# Supplementary material for: A rapid and sensitive method for the simultaneous analysis of aliphatic and polar molecules containing free carboxyl groups in plant extracts by LC-MS/MS
Source: Plant Methods. 2009 Nov 25;5:17. doi: 10.1186/1746-4811-5-17 (PMC2787500; doi:10.1186/1746-4811-5-17)
Supplement: Additional file 1 — Table S1: Coefficients of variation (CV) of replicate measurements for standard mixtures at different concentrations and leaf sample. Table S2. Analysis of derivatized N.attenuata leaf extracts after multiple extractions and calculation of recovery rates. Table S3: Analysis of different amounts of N. attenuata leaf material. [file 1746-4811-5-17-S1.PDF]

Table S1: Coefficients of variation (CV) of replicate measurements for standard mixtures at different concentrations and leaf sample.

Number	Substance	CV (calibration standards)*						CV (leaf sample)*	
		100 pg/µL	250 pg/µL	375 pg/µL	500 pg/µL	750 pg/µL	1000 pg/µL	Intial analysis	analysis after 2 days
1	$^{13}C_6$-jasmonic acid-isoleucine ($^{13}C_6$-JA-Ile)	0.091	0.068	0.054	0.041	0.039	0.031	0.043	0.061
2	Jasmonic acid-isoleucine (JA-Ile)	0.097	0.072	0.048	0.034	0.045	0.033	0.066	0.072
3	$^2H_4$-salicylic acid ($^2H_4$-SA)	0.092	0.037	0.035	0.035	0.04	0.025	0.027	0.045
4	Salicylic acid (SA)	0.091	0.089	0.056	0.031	0.043	0.022	0.043	0.059
5	$^2H_6$-abscisic acid ($^2H_6$-ABA)	0.058	0.047	0.036	0.036	0.06	0.019	0.048	0.051
8	Abscisic acid (ABA)	0.037	0.027	0.033	0.02	0.056	0.014	0.041	0.049
7	Indole-3-carboxylic acid (ICA)	0.094	0.093	0.066	0.052	0.041	0.017	-	-
8	Royal jelly acid (Tr IS)	0.088	0.076	0.031	0.026	0.063	0.037	0.045	0.047
9	Cinnamic acid (CA)	0.048	0.043	0.034	0.015	0.053	0.008	-	-
10	Jasmonic acid (JA)	0.04	0.071	0.031	0.018	0.047	0.006	0.017	0.015
11	Traumatol	0.097	0.091	0.061	0.013	0.01	0.018	-	-
13	Indole-3-acetic acid (IAA)	0.084	0.099	0.088	0.052	0.051	0.035	-	-
14	Traumatin	0.096	0.094	0.067	0.054	0.021	0.021	0.091	0.087
15	9,10-$^2H_2$-dihydro-jasmonic acid (D_2-JA)	0.048	0.06	0.032	0.025	0.046	0.009	0.021	0.022
16	Traumatic acid	0.081	0.073	0.055	0.046	0.039	0.023	0.062	0.068
17	Hexadecatrieonic acid (16:3)	0.051	0.09	0.014	0.018	0.05	0.006	0.087	0.082
18	(9S, 13S)-12-oxo-phytodienoic acid (OPDA)	0.048	0.074	0.062	0.045	0.044	0.021	0.045	0.098
19	Hexadecadienoic acid (16:2)	0.083	0.081	0.054	0.033	0.039	0.024	-	-
20	Gibberellin A_3 (GA_3)	0.086	0.068	0.059	0.047	0.043	0.034	-	-
12	$^2H_2$-OPC 8:0	0.098	0.096	0.068	0.052	0.052	0.021	0.018	0.024
21	Linolenic acid (18:3)	0.054	0.08	0.031	0.037	0.043	0.025	0.053	0.048
22	Hexadecenoic acid (16:1)	0.095	0.096	0.074	0.049	0.051	0.036	0.072	0.058
23	Heptadecenoic acid (17:1)	0.06	0.063	0.046	0.053	0.046	0.018	0.016	0.016
24	Linoleic acid (18:2)	0.087	0.081	0.069	0.061	0.051	0.038	0.05	0.043
25	Hexadecanoic acid (16:0)	0.089	0.011	0.05	0.031	0.061	0.03	0.021	0.024
26	Octadecenoic acid (18:1)	0.095	0.092	0.081	0.036	0.012	0.011	0.028	0.031
27	Heptadecanoic acid (17:0)	0.055	0.061	0.041	0.06	0.041	0.016	0.01	0.011
28	Stearic acid (18:0)	0.044	0.08	0.073	0.061	0.036	0.023	0.007	0.015

* CVs were calculated based on 10 injections for each sample.

Table S2. Analysis of derivatized *N.attenuata* leaf extracts after multiple extractions and calculation of recovery rates (*n* =10)

Substance	control ng gFW^{-1} (±SD)		W+FAC (60 min) ng gFW^{-1} (±SD)		Recovery rates [%]
JA-Ile	-		**114**	(± 9.8)	98.3
SA	**184**	(± 15)	**153**	(± 13)	98.2
ABA	**233**	(± 14)	**264**	(± 11)	98.7
ICA	-		-		
CA	-		-		
JA	**295**	(± 22)	**4380**	(± 67)	99.5
Traumatol	-		-		
dnOPDA	-		-		
IAA	-		-		
Traumatin	**11.3**	(± 0.6)	**17.7**	(± 0.9)	100
Tr. acid**	-		**25.2**	(± 1.8)	100
16:3	**29.6**	(± 2.4)	**41.4**	(± 3.5)	100
OPC-4:0	-		**13,1**	(± 1.0)	100
OPC-6:0	-		**18,9**	(± 1.5)	100
OPDA	**99.3**	(± 6.7)	**254**	(± 14)	97.6
16:2	-		-		
GA_3	-		-		
OPC-8:0	-		-		
GA_4	-		-		
18:3	**445**	(± 21)	**399**	(± 13)	99
16:1	**44**	(± 2.5)	**79**	(± 6)	100
18:2	**253**	(± 21)	**297**	(± 19)	98.4
16:0	**19300**	(± 1800)	**16800**	(± 1300)	99.3
18:1	**638**	(± 51)	**705**	(± 35)	98.6
18:0	**57600**	(± 2800	**47200**	(± 1200)	

**: Traumatic acid

Table S3: Analysis of different amounts of *N. attenuata* leaf material (*n*=2)

	**mass leaf tissue**													
	5 mg		10 mg		30 mg		50 mg		100 mg		150 mg		200 mg	
**Substance**	**Total amount obtained [ng]**													
JA-Ile	-		-		-		**9.14**	(± 0.27)	**17.5**	(± 1.0)	**24.8**	(± 0.32)	**35.6**	(± 1.8)
SA	**0.92**	(± 0.15)	**2.16**	(± 0.28)	**6.26**	(± 0.69)	**7.5**	(± 0.19)	**14.4**	(± 0.6)	**17.5**	(± 2.0)	**26.7**	(± 0.3)
ABA	**0.94**	(± 0.003)	**2.95**	(± 0.15)	**7.4**	(± 0.58)	**15.2**	(± 1.6)	**30.4**	(± 3.6)	**42.7**	(± 1.7)	**62.1**	(± 5.7)
ICA	-		-		-		-		-		-		-	
CA	-		-		-		-		-		-		-	
JA	**62.0**	(± 0.2)	**83.0**	(± 18)	**123**	(± 13)	**229**	(± 21)	**540**	(± 4.5)	**748**	(± 8)	**949**	(± 15)
Traumatol	-		-		-		-		-		-		-	
dnOPDA	-		-		-		-		-		-		-	
IAA	-		-		-		-		-		-		-	
Traumatin	-		-		-		-		-		**1.67**	(± 0.08)	**2.71**	(± 0.18)
Tr. acid**	-		-		-		-		-		**3.0**	(± 0.14)	**4.44**	(± 0.47)
16:3	**0.73**	(± 0.10)	**1.38**	(± 0.07)	**1.53**	(± 0.05)	**3.12**	(± 0.19)	**6.37**	(± 0.11)	**9.02**	(± 1.22)	**16.8**	(± 0.98)
OPC-4:0	-										**3.01**	(± 0.13)	**3.61**	(± 0.09)
OPC-6:0	-										**1.17**	(± 0.10)	**2.38**	(± 0.15)
OPDA	**1.32**	(± 0.58)	**2.96**	(± 0.23)	**9.21**	(± 0.44)	**13.1**	(± 1.42)	**33.0**	(± 6)	**49.2**	(± 6.7)	**65.1**	(± 0.5)
16:2	-		-		-		-		-		-		-	
GA_3	-		-		-		-		-		-		-	
OPC-8:0	-		-		-		-		-		-		-	
GA_4	-		-		-		-		-		-		-	
18:3	**5.16**	(± 0.32)	**7.13**	(± 1.41)	**9.92**	(± 0.07)	**21.1**	(± 1.73)	**39.3**	(± 0.1)	**60.9**	(± 7.3)	**79.1**	(± 0.7)
16:1	**1.5**	(± 0.017)	**3.26**	(± 0.48)	**4.34**	(± 0.12)	**6.82**	(± 0.50)	**12.7**	(± 0.3)	**18.9**	(± 1.6)	**25.6**	(± 0.5)
18:2	**5.35**	(± 0.006)	**9.33**	(± 1.23)	**16.5**	(± 2.3)	**19.2**	(± 1.45)	**36.4**	(± 1.4)	**56.7**	(± 4.7)	**71**	(± 1.4)
16:0	**57**	(± 13)	**140**	(± 8)	**470**	(± 40)	**868**	(± 15)	**1608**	(± 151)	**2258**	(± 61)	**2949**	(± 131)
18:1	**3.34**	(± 0.56)	**5.46**	(± 0.51)	**8.16**	(± 0.91)	**20.8**	(± 2.5)	**58**	(± 4)	**82.7**	(± 3.8)	**114**	(± 8)
18:0	**209**	(± 37)	**463**	(± 12)	**731**	(± 2)	**2178**	(± 76)	**4476**	(± 168)	**6070**	(± 34)	**8705**	(± 69)

**: Traumatic acid
